# The Influence of the Hybridization Process on the Mechanical and Thermal Properties of Polyoxymethylene (POM) Composites with the Use of a Novel Sustainable Reinforcing System Based on Biocarbon and Basalt Fiber (BC/BF)

**DOI:** 10.3390/ma13163496

**Published:** 2020-08-07

**Authors:** Jacek Andrzejewski, Bartosz Gapiński, Aminul Islam, Marek Szostak

**Affiliations:** 1 Institute of Materials Technology, Polymer Processing Division, Faculty of Mechanical Engineering, Poznan University of Technology, Piotrowo 3 Street, 61-138 Poznan, Poland; marek.szostak@put.poznan.pl; 2 Institute of Mechanical Technology, Faculty of Mechanical Engineering, Poznan University of Technology, Piotrowo 3 Street, 61-138 Poznan, Poland; bartosz.gapinski@put.poznan.pl; 3 Department of Mechanical Engineering, Technical University of Denmark, Produktionstorvet, Building 427A, 2800 Lyngby, Denmark

**Keywords:** hybrid composite, basalt fiber (BF), biocarbon (BC), mechanical performance, structure orientation

## Abstract

The presented work focuses on the assessment of the material performance of polyoxymethylene (POM)-based composites reinforced with the use of a biocarbon/basalt fiber system (BC/BF). The use of BC particles was aimed at eliminating mineral fillers (chalk, talc) by using fully biobased material, while basalt fibers can be considered an alternative to glass fibers (GF). All materials were prepared with the same 20% filler content, the differences concerned the (BC/BF) % ratio. Hybrid samples with (25/75), (50/50), and (75/25) ratios were prepared. Additionally, reference samples were also prepared (POM BC20% and POM BF20%.). Samples prepared by the injection molding technique were subjected to a detailed analysis of mechanical properties (static tensile and Charpy impact tests), thermomechanical characteristics (dynamic mechanical thermal analysis—DMTA, heat deflection temperature - HDT), and thermal and rheological properties (DSC, rotational rheometer tests). In order to assess fiber distribution within the material structure, the samples were scanned by a microtomography method (μCT). The addition of even a significant amount of BC particles did not cause excessive material brittleness, while the elongation and impact strength of all hybrid samples were very similar to the reference POM BF20% sample. The tensile modulus and strength values appear to be strictly dependent on the increasing BF fiber content. Thermomechanical analysis (DMTA, HDT) showed very similar heat resistance for all hybrid samples; the results did not differ from the values for the POM BF20 sample.

## 1. Introduction

The main objective of this research is to develop sustainable polymer matrix composites and to test the feasibility of using biomaterials as a replacement for traditional synthetic filler materials. The results of this work offer more environmentally friendly options for composite materials for high-end engineering applications. One of the biobased fillers used in this investigation is biocarbon. The second filler component was basalt fiber in the form of chopped filament strand. The basalt fibers acting as a reinforcement in the composite materials can be treated as a more sustainable alternative to the glass fiber reinforcement due to the less material and energy-consuming manufacturing process. At present, there are no comprehensive studies on the life cycle assessment (LCA) of polymer composites with the addition of basalt fibers, which also applies to available LCA databases. However, the analyses conducted for the construction industry indicate a much lower environmental impact of the BF product manufacturing process in relation to glass fiber or steel-reinforced composites [1,2,3]. According to Gkaidatzis [4], the total energy required for basalt fiber production is 17.85 GJ/tonnes, while the same requirements for glass fibers can reach 54.7 GJ/tonnes.

Previous studies have repeatedly discussed the use of both types of fillers individually; however, hybrid systems based on BC and BF are described for the first time in the current paper. There are also no literature reports on the use of these types of fillers for POM. Therefore, the presented investigation is important for the future use of sustainable reinforcing systems in the processing of technical polymers. Previous research on the use of biochar-based composites concerned mostly commodity plastics and low-end applications, while the applications for POM are associated with technical products with very high requirements in terms of precision and performance. For this reason, the results and analysis presented in this paper will be valuable for the production and processing of sustainable composite materials for highly demanding industrial applications.

Taking into account the global demand for polyoxymethylene, the consumption of this polymer is relatively low, even considering only the category of technical polymers. According to data from the Global Market Insights portal, the global consumption of polyacetals reached 1.2 million tonnes in 2016. It is expected that by 2024, the average annual increase in the POM market will be 8.8%, reaching about 8000 million USD [5,6] For comparison, the consumption of polyamides is about 7.4 million tonnes (PA6 and PA66) [7]. It is worth noting, however, that in the case of polyamides, fiber production has a large share of the market, while for POM, resin injection molding is the most popular processing method, especially the production of precise products. This is mainly due to excellent dimensional stability, high thermomechanical resistance, and a low friction coefficient. High heat resistance is one of the key reasons for using POM. This polymer has a very high level of crystallinity, usually exceeding 50% [8,9]. The high thermal stability of the POM crystalline structure is confirmed even for pure POM samples, while the HDT parameter values exceeds 135 °C, which is only 30 °C less than the melting point of POM. For comparison, polypropylene (PP), whose melting point temperature (≈165 °C) is very close to that of POM, the HDT value reaches around 80–110 °C [10,11,12]. These features are strongly modified in the case of materials modified with fibrous reinforcement. In the case of precision products, it is particularly important to maintain a high accuracy of molding geometry, which is why fiber-reinforced materials are not always applicable, due to the structure anisotropy. The presented work might contribute to the production of functionally versatile POM composites and make the use of POM material more widespread by elimination some of the existing problems with composite materials.

The phenomenon of fiber orientation during the injection molding process is the main reason for the structure anisotropy of composite materials. It is also the key issue in the context of dimensional accuracy required for molded products, which is especially important when manufacturing precision products such as gears, threads or plugs. One of the possible solutions to this problem is the use of a hybrid filler system [13,14]. Spherical particles introduced into the composite system cause reorganization of the polymer flow structure. The natural tendency of fiber arrangement along the direction of shear forces is disturbed, which results in greater randomization of the fiber position during the polymer flow, and consequently in the finished product. Hybrid composites have been known for many years; their industrial application is also quite common [15,16,17]. As an example, there can be used materials reinforced with glass fibers and a powder additive in the form of powdery fillers [18,19]. Materials of this type have been used for many years on an industrial scale, mainly in combination with technical polymers such as PA6, polybutylene terephthalate - PBT or POM [20,21,22]. Despite the fact that the subject of the use of biocarbon fillers has been studied for many years, the BC-based composite materials are most often compared with classic fillers (GF, talc, chalk), and the research works devoted to the hybrid system containing biocarbon are quite rare. The research of Abdelwahab et al. is an example of the use of BC and a nanoclay system [23], where the use of a hybrid system resulted in an improvement of the mechanical properties and dimensional stability. Another example is the research on the use of a biocarbon/carbon fiber system by Matykiewicz [24], where BC was used as a low-cost filler for epoxy-based laminates. Other studies are more focused on comparing BC-filled systems with different types of reinforcement.

Biocarbon (BC) in the powder form proves to be a valuable substitute for conventional mineral fillers, such as chalk, talk, and other powdery fillers. In contrast, BC has a much lower density than mineral fillers, which in real applications may translate into a lower weight of manufactured products. This was already proved for several types of polymers such as polypropylene [25,26,27,28,29], nylon [30,31,32] or polyesters [33,34,35]. The use of biocarbon in the plastics processing industry is currently one of the new development directions for composites reinforced with natural fillers. Biocarbon, which is a product of biomass treatment in the pyrolysis process, is characterized by high thermal stability, which allows the use of engineering polymers in processing, even above 250 °C, which is impossible for classic lignocellulose fillers. In the case of planned tests, the source of biomass will be the waste of the wood industry in the form of wood chips.

Basalt fibers (BF) were the second key material used during the research. This material is now increasingly used, especially in technical applications, where until now mainly glass fibers (GF) have been used. The properties of basalt fibers do not differ significantly from those of the GF, because the slightly higher density of basalt is compensated for by higher strength. Similar to glass fibers, the basic component of basalt is also silica; however, the exact composition of basalt fibers is more complex. Most commonly, the basalt fiber composition is as follows: SiO_2_–52.8%, Al_2_O_3_–17.5%, Fe_2_O_3_–10.3%, MgO–4.63%, CaO–8.59%, Na_2_O–3.34%, K_2 ball mill_O–1.46%, TiO_2_–1.38%, P_2_O_5_–0.28%, MnO–0.16%, Cr_2_O_3_–0.006%. It is worth noting that the chemical content may differ based on the geographical origin of the basalt deposits [36,37,38]. The main advantage of basalt fibers is their production process eliminating the use of alkali glass. The input product in the process of melting and spinning fibers is basalt rock processed into melt at a temperature of about 1500 °C. The production of basalt products can therefore be called a one-stage process, which eliminates many negative aspects of the production of other types of mineral fibers.

The subject of this research is the examination of the new variety of hybrid composites reinforced with biocarbon (BC) and basalt fibers (BF). Neither of these materials has been used in such a system so far. The conducted research allows us to evaluate many practical aspects of the use of biocarbon and its hybrid systems reinforced with basalt fibers.

## 2. Materials and Methods

### 2.1. Materials

Tarnoform 300 acetal copolymer was used for the purpose of this study; this type of injection molding grade of POM resin is produced by Grupa Azoty (Tarnow, Poland); the melt flow index (MFI) of this type of POM is 9 g/10 min (2.16 kg/190 °C). The basalt fibers were in the form of chopped strands, length of 3.2 mm and diameter of 13 μm. The material was produced by Kamenny Vek Co. (Moscow, Russia) and supplied by TechSolutions (Skarzysko Kamienna, Poland).

**Figure 1:**
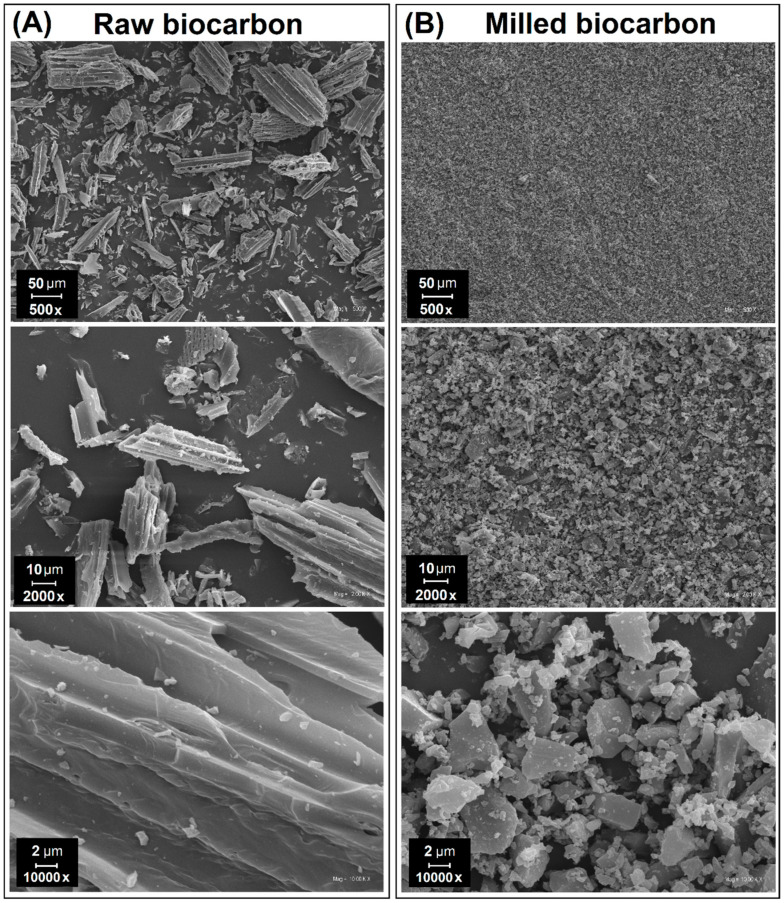
Biocarbon particles (**A**) before and (**B**) after the ball milling procedure (24 h).

Biocarbon used for this research was prepared from commercially available biochar produced by the company Fluid S.A. (Sedziszow, Poland). This material was obtained from pyrolyzed biomass, in this case wood chips. The maximum temperature of the pyrolysis process is 650 °C. Because the initial grain size for supplied biocarbon can be reached even at 10 mm, ball milling was used to reduce the particle size. The used machine was a BK2 type ball mill (Metalchem, Łódź, Poland); the milling time was set to 24 h, after which the particle size was reduced below 10 μm, which is shown in Figure 1.

### 2.2. Sample Preparation

Composite mixtures were prepared by melt blending using a twin screw extruder Zamak EH-16.2 D (Zamak Mercator, Skawina, Poland). The maximum temperature of the extrusion process was set to 190 °C for all POM-based composites. A ccrew speed of 100 rpm was also constant during the whole procedure. Before processing, POM pellets were dried in a cabinet drier to reduce the moisture content; the procedure was performed in 80 °C for 12 h. A similar procedure was applied to the biocarbon powder and basalt fibers; however, the drying conditions were modified to 110 °C and 48 h. Before pouring into the extruder feeder, all ingredients were dry blended in a plastic bag. For all types of polymer composites, the filler content was 20% measured by weight. Individual sample names were marked by indicating the biocarbon/basalt fiber ratio (BC/BF). For example, the POM (50/50) sample was prepared from polyoxymethylene resin with the addition of 10 wt.% biocarbon and 10 wt.% basalt fibers.

ENGEL e-mac 50 (Engel GmbH, Schwertberg, Austria) was the injection molding machine used during the sample preparation stage; the injection molding temperature was set to 200 °C, while the mold temperature was 80 °C. The injection rate was 35 cm^3^/s and the injection pressure was 1050 bar while the holding pressure was reached. The holding time was set to 10 s, while the cooling stage time was 20 s. Fixed processing parameters were used for all material formulations; the detailed list of parameters is shown in Table 1.

### 2.3. Characterization

The universal testing machine model Zwick/Roell Z020 (Zwick Roell GmbH, Ulm, Germany) was used to perform the static tensile tests. The measurements were conducted according to the ISO 527 standard [39]; for all samples, a cross-head speed of 5 mm/min was used. The notched Charpy method (ISO 179 standard [40]) was implemented to investigate the impact resistance of the samples; the used hammer type was a Zwick/Roell HIT25 attached to a 5 J pendulum.

In order to conduct the DMTA analysis, we used the Anton Paar MCR 301 apparatus (Anton Paar GmbH, Graz, Austria); the machine was attached with a torsion mode fixture, and the dimension of all samples was similar, 50 × 10 × 4 mm. The measurements were conducted from 30 to 150 °C, and the heating rate was 2 °C/min. The constant deformation amplitude (strain) was set to 0.01%, and the deformation frequency was 1 Hz. Storage modulus and tan δ results were collected and presented in the form of thermogram plots.

The heat resistance was investigated using two testing methods. The heat deflection temperature (HDT) test was the first method. Measurements were conducted according to the ISO 75 standard [41], where the heating rate was set to 2 °C/min and the load was 0.455 MPa the span distance was 64 mm (flatwise test). The Vicat softening temperature (VST) measurement was performed as the second type of measurement, where a 4-mm-thick specimen was prepared to conduct the test; the whole procedure was performed in accordance with the ISO 306 standard [42]: heating rate of 2 °C/min and applied force of 10 N. For both types of measurements, an RV300C HDT/Vicat machine was used (TestLab, Warszawa, Poland); this apparatus was equipped with an oil bath.

Thermal properties of the prepared samples were investigated by means of the differential scanning calorimetry (DSC) method. The apparatus used during the study was a DSC 204 F1 Phoenix (Netzsch-Gerätebau GmbH, Selb, Germany). The tests were performed under a protective atmosphere of nitrogen, while during the test, the samples were stored inside aluminum crucibles. The temperature program consisted of two heating stages, from 20 to 220 °C at a heating rate of 10 °C/min; after the first heating stage, the sample was cooled at the same rate of 10 °C/min. In order to calculate the crystallinity level, we used the following equation:

(1) $$\%\;Crystallinity=X_{c}=100\times \frac{\Delta H_{m}}{\Delta H_{POM}\;\left(1-\varphi \right)}$$

where Δ*H_m_* is the melting enthalpy measured from the heating scan, Δ*H_POM_* represents the melting enthalpy of 100% crystalline POM (186 J/g) [8,43]; the weight fraction of the filler is expressed by φ.

Density measurements of the molded samples were conducted using the immersion method (ISO 1183 standard [44]). We used an Axis AD2000 precision balance equipped with a special adapter. For basalt fibers and biocarbon particles, the density measurements were conducted according to the ISO 12154standard [45] using a helium pycnometer (Thermo Scientific Pycnomatic). This method was helpful to determine the porosity/void content of the prepared samples.

An Anton Paar MCR 301 rotational rheometer (Anton Paar GmbH, Graz, Austria) was used to perform the rheological characterization. Small angle oscillation tests were conducted with the use of plate-plate geometry, with a 1 mm gap, and a 25 mm plate diameter. The initial amplitude sweep tests we conducted in order to determine the linear visco-elastic region (LVE) of the tested materials. The second stage of characterization consisted of frequency sweep measurements. A constant strain of 0.5% was used for all measurements; the deformation frequency ranged from 0.1 to 100 rad/s. All test were conducted at 190 °C, under a protective atmosphere of nitrogen. Flat cylindrical samples (diameter = 25 mm, height = 2 mm) were used to perform the tests; specimens were prepared during the injection molding stage.

Thermal stability/degradation of the prepared samples was investigated with the use of thermogravimetric analysis (TGA). A precise balance model Libra 209 F1 (Netzsch, Selb, Germany) was used during the study. The measurement temperature range was set to 30–800 °C, and the heating rate to 10 °C/min. All measurements were performed under a protective nitrogen atmosphere. The average size of the sample was 10 mg.

Scanning microscope analysis (SEM) was used to conduct the structure evaluation. The observed surface was cryo-fractured after immersion in liquid nitrogen; after that, the surface was coated with a conductive layer of gold. An EVO 40 SEM microscope (Carl Zeiss AG, Jena, Germany) was used to conduct the analysis.

The structure of the composite samples was examined with the use of a measuring X-ray tomograph, model v|tome|x s240 (Waygate Technologies / GE Sensing & Inspection Technologies GmbH, Wunstorf, Germany). The use of μCT was focused on the evaluation of the structure orientation [46], while usually this type of machine is used for metrological study [47]. Following scanning, parameters were used during the measurements—nanofocus x-ray tube (voltage 100 kV/current 200 μA); the exposure time for one picture was 500 ms, and the voxel size was 8.5 μm for thin samples and 11 μm for thick ones. The histograms presenting the composite structure orientation were prepared on the basis of image analysis performed using ImageJ (1.53a version) software. Basalt fiber arrangement was analyzed using the OrientationJ plugin, where the task of this software is to characterize the orientation in the image based on the evaluation of the structure tensor in the local neighborhood. In order to eliminate the noise resulting from the presence of biocarbon particles and the background of the image itself, image processing was applied, and a binary object mask was created. This procedure was developed to assess the orientation of tissue structures such as collagen fibers [48,49,50], while it is often used for evaluation of the fiber arrangement in polymer composites [51,52,53], which is also the subject of the presented research.

The full list of samples is presented in Table 2; in addition to the designation of samples and the composition of individual materials (weight and volume fraction), the table also contains the results of density measurements together with porosity calculation.

## 3. Results and Discussion

### 3.1. Mechanical Performance—Static Tensile Tests and Impact Resistance Measurements

**Figure 2:**
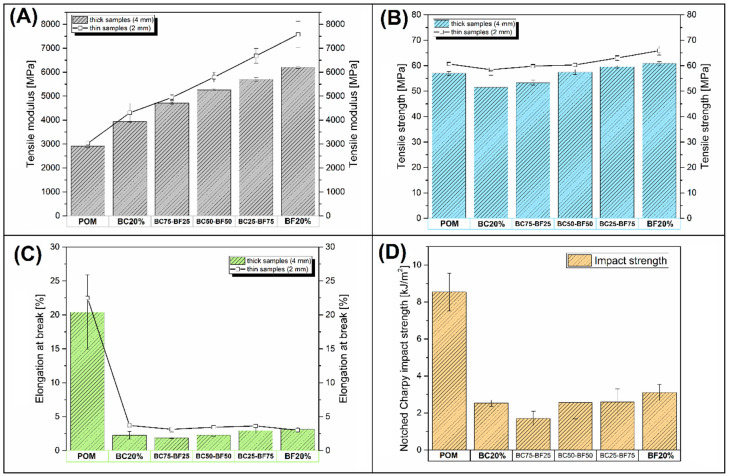
The results of the mechanical tests of the prepared composites: (**A**) tensile modulus; (**B**) tensile strength; (**C**) elongation at break; (**D**) notched Charpy impact strength.

The results of the mechanical test are presented in Figure 2; the particular charts summarize the obtained values of tensile modulus, tensile strength, elongation at breakage, and impact strength. For tensile tests, results were obtained for both thick (4 mm) and thin (2 mm) samples, while the Charpy test was performed only for standard thick ISO samples (80 × 10 × 4 mm). As predicted, the stiffness of composite samples was strongly improved after the introduction of the fillers; moreover, the reinforcing efficiency was directly related to the basalt fiber content. The initial tensile modulus for the pure POM sample was around 2900 MPa; the addition of BC particles (20%) increased the modulus to 3935 MPa. At the same 20% content of BF, the tensile modulus reached around 6200 MPa. The stiffness of individual hybrid composites had intermediate values depending on the fiber content. The results for thin 2 mm samples were slightly higher than the modulus values obtained for standard 4 mm ISO specimens; however, as the fiber content increased, the difference in favor of thinner samples increased. This behavior is due to the greater orientation of thin-walled products. For tensile strength values, the analysis revealed that introduction of the filler did not deteriorate POM properties significantly. The highest strength reduction was observed for the POM/BC20 composite; however, the strength decrease was small, from 57 MPa for pure POM to 51.5 MPa after introduction of the BC filler. The addition of basalt fibers in hybrid composites caused a gradual increase in strength, up to 61 MPa for pure BF-reinforced composites. A difference in the tensile strength value between thick and thin samples was observed again; however, this time it was difficult to find a visible trend of these changes. The most significant deterioration of properties was observed for the elongation at breakage results. The initial strain for pure POM was around 20%, while the addition of the fillers decreased that value to around 3%. Similarly, for tensile modulus and strength values, the results of elongation were also slightly higher for thin-walled samples. Impact resistance was evaluated using the notched Charpy method. The analysis of the obtained results revealed a large decrease in the impact strength; the results are in line with elongation at breakage value changes.

Considering the results for other types of biocarbon-reinforced composites, it can be safely stated that the results obtained for POM are very promising. As the main example, we can cite research on hybridization of PC-based composites with the addition of a biocarbon/carbon fiber system [54], where the presence of BC particles in each case decreases the mechanical properties, while the addition of carbon fibers only compensated for the deterioration of material parameters. For polycarbonate, however, the authors confirmed that a serious reason limiting the use of powder fillers is high sensitivity to the moisture usually present in biocarbon. Slightly better results of biochar addition are therefore observed for thermoplastic polyesters. The research works on the use of BC in composites based on PBT [34], polyethylene terephthalate—PET [55], and poly(lactic acid)—PLA [35,56] indicate that the reinforcing efficiency for talc is only slightly worse than that for talc particles. So far, the best effects on materials with the addition of BC were observed for nylons [32,57,58]. However, it is worth pointing out that, as in the case of the presented research study, literature examples indicate the need for hybridization of BC/composites using fibrous reinforcement.

**Figure 3:**
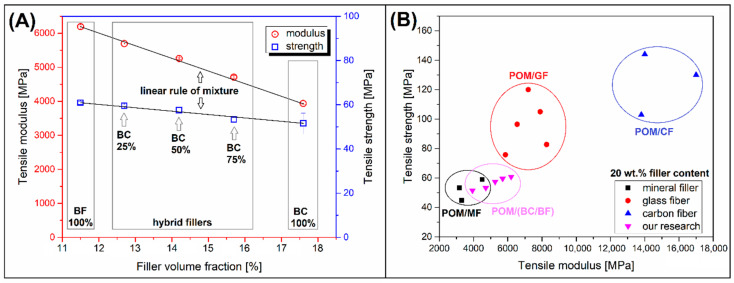
The analysis of reinforcing efficiency: (**A**) role of mixture (ROM) fitting for hybrid composites; (**B**) Ashby plot presenting the comparison of commercial-grade composites and obtained materials.

For a more detailed analysis of the effectiveness of the applied reinforcement system, an analysis of the modulus and strength values was prepared (see Figure 3A). The plots were presented as a function of the filler volumetric content (fraction), so that it is possible to determine whether the change trends are consistent with the simple rule of the mixture model (ROM). Typically, the matching of the composite property changes to the ROM model is analyzed for compositions containing different filler contents, which is somewhat difficult due to the use of a fixed 20 wt.% filler content in the presented study. However, due to significant differences in BC and BF densities and the resulting differences in the volume content of the filler system, it is possible to determine a simple dependeny for modulus and strength. The chart clearly shows that the changes in the values for both the modulus and the strength are almost linearly consistent with the ROM model, which indirectly confirms that the addition of biochar does not cause an excessive reduction in the mechanical properties of the material. Apart from the obvious decrease of the structure stiffness after the addition of BC, it is worth noting an additional positive aspect related to a slight change in tensile strength. The positive aspect of the entire procedure is also the increase in the volume fraction of fillers after the addition of BC particles, which in the case of commercial applications may contribute to lowering the density of composites and reducing the weight of the final products.

A simple comparison of the composites prepared in the presented research with commercially available materials is presented in Figure 3B where the Ashby-type plot is presented. As part of the comparison, we have included tensile modulus and strength data for several commercially available polyoxymethylene-based composites (see Table 3). We chose the most common types of fillers in the form of mineral filler, glass fiber, and carbon fiber; for all materials, the weight content of the filler was 20%. There is no doubt that the best modulus and strength proportions were obtained for CF-based materials, while the mineral fillers (MF) can be consider the least effective reinforcing systems. The tensile modulus of the prepared materials was slightly higher than that obtained by POM/MF materials and reached the value obtained by some POM grades with the addition of GF. Unfortunately, the tensile strength of GF-reinforced materials was much higher than the values for exanimated POM hybrids. The reason for such a low efficiency is the lack of an appropriate surface compatibilizer (sizing agent), which was confirmed by SEM photos.

### 3.2. Heat Resistance—DMTA Analysis and Heat Deflection Measurements

**Figure 4:**
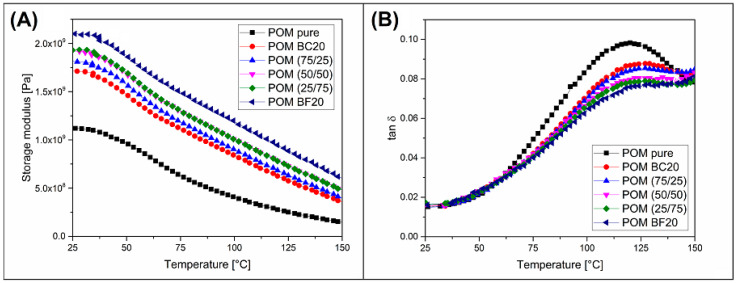
The comparison of the storage modulus (**A**) and tan δ (**B**) plots for different types of POM-based composites.

Changes in HDT values are usually closely related to differences in material stiffness on a temperature scale. DMTA analysis turns out to be a very helpful tool in analyzing the impact of the hybridization method on the thermomechanical properties of composite materials. The test results for all POM-based samples are visualized in Figure 4. The graphs show the measurements of the storage modulus and the tan δ values. It is easy to conclude from the storage modulus plots that the stiffness of all types of composite samples is visibly improved in comparison to pure POM resin. It is also clear that the increasing content of the basalt fiber in the structure translates into the higher stiffness measured by the value of the storage modulus. It is worth mentioning that a slight falling curve profile for POM-based samples is an additional confirmation of the thermal stability of the material structure. Rapid changes in the course of the storage modulus curve are evidence of phase transitions, most often the glass transition of the polymer amorphous phase, and it is especially noticeable for low crystalline thermoplastic polyesters (PLA, PBT or PET) [59,60,61,62]. A typical feature of this type of material is the direct link between the HDT/Vicat temperature range and the decrease in storage modulus around T_g_. For highly crystalline materials, the effect of the glass transition of the amorphous phase is less significant, which is reflected in the stable course of the storage plot. Polyolefins or certain types of polyamides exhibit similar behavior to POM resin [57,63,64]. The thermomechanical stability of POM samples is confirmed by the tan δ plots, where for the entire temperature range the curves did not indicate significant changes in material dumping behavior.

Results of stiffness measurements in the static tensile test and during DMTA analysis indicate different trends for hybrid materials. In the case of static tests, the addition of basalt fibers suggests a higher structure reinforcing efficiency, while the results of DMTA tests suggest only small changes. These differences are the result of different geometries of the measurement system for performed measurements. The fiber orientation is more favorable for the tensile test, while for the torsion mode used during the DMTA analysis, the fiber arrangement had less effect on the stiffness results.

The intensity of the polymer-filler interaction can also be assessed using DMTA analysis results. For this purpose, the degree of the entanglement N factor can be calculated. The value of this coefficient is calculated based on the results of storage modulus [65]. This factor has already been used in the assessment of structural phenomena for many varieties of composites [54,66,67,68]. In order to assess the changes in structure interactions, the N factor is determined for several temperatures, so that it is possible to evaluate the interaction differences due to material phase transitions or for different temperatures of composite operations. The following equation was used:

(2) $$N=\frac{G^{\prime }}{6RT}$$

where G′ represents the value of storage modulus at a calculated temperature, R is the universal gas constant, and T is the temperature expressed in Kelvin absolute scale. Table 4 presents the results of the storage modulus values and N factor calculations. The G′ values were recorded at the beginning of the DMTA measurement at 25 °C, and at 135 °C, which was the HDT temperature of the pure POM sample and can be considered an important reference point for composite materials. As could be expected, the N factor value of the POM BC20 composite was the lowest, similar to other direct studies. The gradual increase in density resulting from the addition of basalt fibers was rather small, and even for the POM BF20 sample, does not suggest a significant change in structural interactions. Analyzing the absolute values of the N factor, between the 25 °C and 135 °C temperatures, it should be stated that the change in the interactions confirms the lack of polymer phase changes occurring in the measured range.

HDT temperature measurements clearly reflect the results of DMTA analysis (Table 4). Despite the fact that the thermal resistance for pure POM was very high, surprisingly the addition of biochar in the amount of 20% increased the HDT value by almost 20 °C, which can be considered a significant increase. The introduction of additional basalt reinforcement improved the HDT result; however, the increase in structure reinforcing efficiency was not very high. For the POM BF20 composite, the HDT value reached around 162 °C, which is about 7 °C higher than that for the POM BC20 sample. HDT measurement results largely reflected the differences in storage modulus values for higher temperatures recorded at the end of the DMTA test, which is why the table show the G′ values for 135 °C.

### 3.3. Thermal Behavior—DSC Analysis

**Figure 5:**
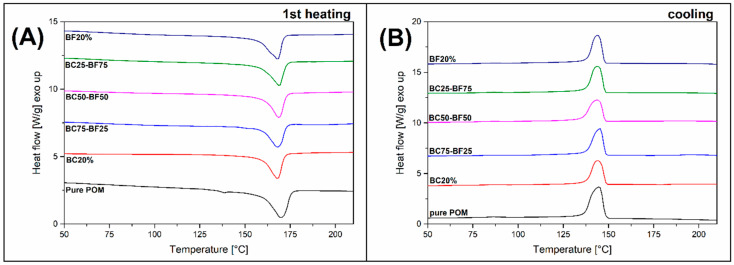
The results of DSC analysis in the form of thermograms. (**A**) 1st heating stage and (**B**) cooling stage signals.

Most of the conducted research indicates a very important role of the high degree of POM crystallinity on the properties of prepared polymer composites. The main purpose of DSC analysis ias to show possible differences in the content of the crystalline phase or its growth kinetics. Some research indicates that for some types of polymer fillers, an increase in mechanical properties is possible through changes in the POM crystalline phase morphology [69]; this is particularly visible for polymer nanofillers [8,70]. Thermograms showing the DSC signal for POM (BC-BF) composites are presented in Figure 5, both for 1st heating and cooling scans. Table 5 summarizes the basic thermal properties obtained during the DSC test. The results clearly indicate the lack of a visible impact of the used composite fillers on the melting and crystallization processes of the POM structure. The phase transition temperatures did not shift, and the total content of the POM crystalline phase for all materials was about 70%.

It should be added that in the case of polymers with high crystallization kinetics, such as POM, attempts to change the crystalline phase morphology are usually not effective. An example here is not only research on the use of composite fillers [69,71], but also experience in the preparation of polymer blends [72,73,74,75].

### 3.4. Rheological Characteristic—Small Amplitude Oscillation Shear Measurements (SAOS)

**Figure 6:**
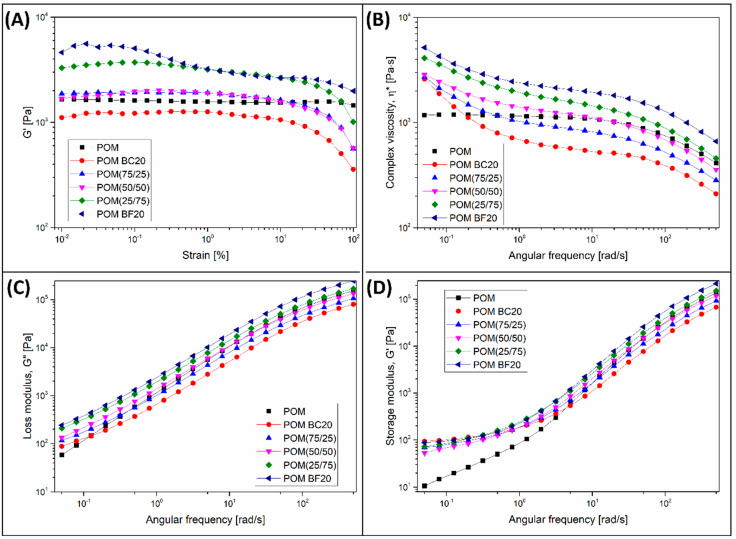
The results of rheological analysis performed using the rotational rheometer: (**A**) G′ plots from amplitude sweep tests; (**B**) complex viscosity η*; (**C**) loss modulus G′′, and (**D**) storage modulus G′.

Rheological characteristics of the tested composites were analyzed during tests carried out using a rotational rheometer. In order to determine the linear viscoelastic region (LVR), the first phase of the measurements was performed in amplitude sweep mode; after that, the frequency sweep tests were carried out. The results of both tests are presented in Figure 6, where G′/strain plots from the amplitude sweep measurement were collected with the results of frequency sweep tests: complex viscosity η*, loss modulus G′′, and storage modulus G′.

The influence of the filler content can be noted in each of the presented charts. The G′ plots presented in Figure 6A indicate clearly the presence mechanical interactions between filler particles, which is observed for samples with large amounts of BF in the structure, POM (25/75) and POM BF20. The confirmation of this behavior is the visible increase in the G′ at low strain values. This kind of behavior was already reported for GF-based composites [76,77], and confirms that in the case of fibrous structures where the L/d ratio reaches significant values, the presence of physical interactions between the fibers always has a significant impact on the rheological characteristics.

The increase in viscosity caused by the increasing share of BF fibers in the structure is also visible in the graphs comparing the results of frequency sweep tests. This can be seen in the graphs of complex viscosity (Figure 6B), where a strong increase in the η* values is reported for all composite samples, especially at low deformation frequencies. The changes also apply to the loss G′′ and storage G′ modulus values. Despite the fact that during the injection molding tests no visible signs of degradation could be observed, the results of the rheological analysis indicate some visible reduction in the molecular weight of POM resin in the presence of BC particles. The confirmation of the matrix decomposition phenomenon is a significant decrease in viscosity for BC-rich samples, which can be seen in the complex viscosity graph (Figure 5B). For almost the entire measuring range, the values of η* for the POM BC20% and POM (75/25) sample are lower than those for the reference POM. The increase in absolute values of viscosity for the rest of the hybrid samples does not indicate the disappearance of the degradation phenomenon, but only its limitation. This assumption is confirmed by the analysis of G′ in Figure 5A, where the LVR range for all samples containing BC is clearly smaller than for both pure POM and POM BF20% samples.

Each of the prepared composite materials still has a relatively high viscosity; however, this kind of behavior might suggest some negative consequences of using biocarbon. The biocarbon structure has some hygroscopic tendencies due to its high porosity. Even after applying the drying procedure, the moisture content of the material is still about 1%, while before drying, is usually around 7–10% [78,79]. The presence of moisture may lead to the increased intensity of hydrolytic degradation phenomena, which were observed for polycarbonate/biocarbon (PC/BC) composites [54]. POM is a polymer much more resistant to the presence of water during processing. However, taking into account its general tendency for thermal degradation, an additional reason for the decrease in the viscosity of POM BC20% composites in relation to the reference POM sample is the additional processing cycle during the melt blending on a twin screw extruder. The issue of the effects of repeated processing is particularly relevant and has already been the subject of many research works [80,81]. However, in the case of polymer composites, degradation of the polymer structure is only one of the factors determining the final properties, while the decrease in molecular weight is not often decisive.

### 3.5. Thermogravimetric Analysis—TGA Measurements

**Figure 7:**
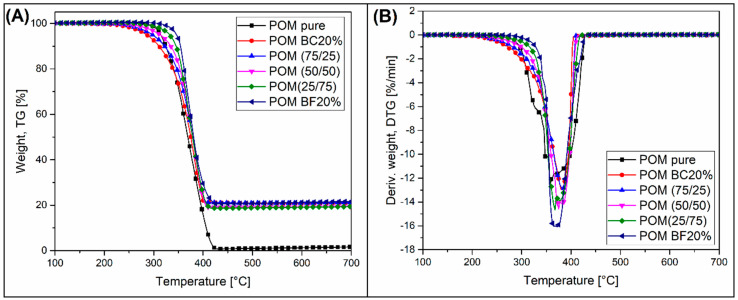
The results of thermogravimetric analysis of POM-based composites. (**A**) TG thermograms and (**B**) DTG thermograms.

The results of the TGA analysis are presented in Figure 7, where thermogravimetry weight loss curves (TG) and derivative of the TG weight loss plots (DTG) are shown. The preliminary analysis clearly shows that the real filler content in the individual samples is very similar and close to 20%. This description does not apply to the POM sample, where the final material content is close to 0%, due to the complete decomposition of the polymer resin. Apart from the results obtained for the reference sample (POM pure), changes in the course of TG thermograms for composite materials show a clear tendency, where the onset of the decomposition temperature is dependent on the BC content. The results confirm that the addition of BC in the structure may have an impact on the intensification of the polymer chain degradation phenomenon, which is also confirmed by rheological analysis. Taking into account the 5% weight loss of the sample, which is usually a determinant of the beginning of the polymer matrix decomposition process, the highest thermal stability of around 350 °C was recorded for the POM BF20% sample. The lowest result for the POM BC20% sample was close to 300 °C. The values for the remaining hybrid composites fell within this range and decreased with the share of the BC filler in the composite structure. Changes in the position of the DTG curve peak were rather insignificant and did not constitute a valuable object of analysis. However, the peak onset value shift, similar to 5% weight loss temperature, suggests a negative influence of BC on the temperature stability of composites.

### 3.6. Structure Evaluation—Scanning Electron Microscopy Observations (SEM)

**Figure 8:**
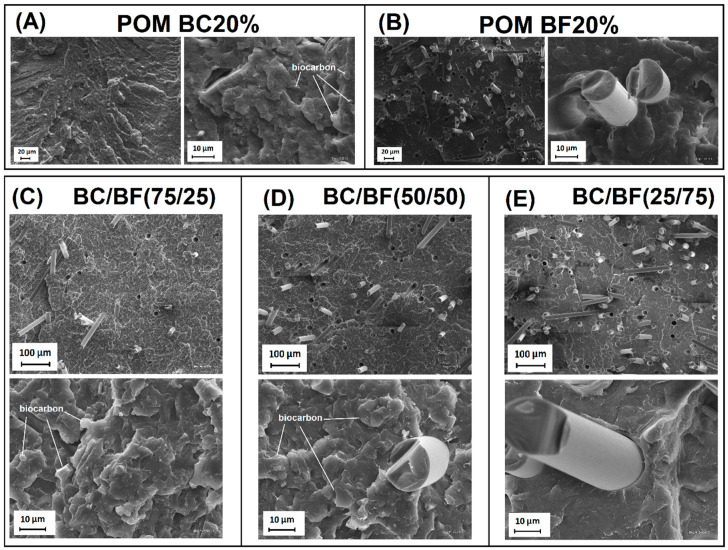
SEM images presenting the fractured surface obtained after the impact test. (**A**) POM BC20%; (**B**) POM BF20%; (**C**) POM (75/25); (**D**) POM (50/50); (**E**) POM (25/75).

SEM structure views for composites prepared during the study can be seen in Figure 8. The images show the fractured surface of samples obtained after the impact tests. It is worth noting that in the case of the POM BC20% composite structure, it is difficult to clearly distinguish the structure of biocarbon particles, which can confirm the high level of adhesion on the BC–POM interface. An important factor determining this type of behavior is the high level of fragmentation of the BC particles that were milled for 24 h. In the case of many previous studies, a large fraction of BC was also used, where the average particle size exceeded 100 μm [11,31,82,83]. For this kind of composite, the microscopic observations allowed for clear separation of the porous structure of the BC filler. However, as other studies indicate [23,56], the milling procedure used during these studies usually gave better results, which is confirmed by the properties obtained for the tested materials. The addition of BF is very clearly visible in the structure, also for hybrid samples containing their small addition, such as for sample POM (75/25) (BF content = 5 wt.%). The BF-POM interface appearance suggests a lack of strong adhesion between the matrix resin and the fiber surface. However, this problem occurs also for many other types of BF/thermoplastic polymer composites [84,85,86].

Summarizing the SEM structural observations, it is worth noting that the appearance of the structure of composites with the addition of BC suggests good matrix-filler compatibility. The presumed reason for high adhesion at the interface is the presence of organic functional groups on the BC surface. These phenomena have already been described many times for composites based on PA6 and PP [11,30,58,83].

### 3.7. Fiber Structure Orientation—X-Ray Microtomography Measurements (μCT)

The μCT scanner measurement allowed us to determine the changes in the orientation of the composite structure. In the case of the discussed measurements, the research methodology was focused on isolating changes in the orientation of BF fibers due to the addition of BC particles. The measurements were carried out on 4 and 2 mm thick dumbbell samples. Internal structure scans were performed in the middle section of the samples. For comparative purposes, cross-sections of the samples were made along the sample’s symmetry axis and at the sample sidewall surface. Such imaging enabled the assessment of changes in the orientation of the material structure, both in terms of changes in the composition of the composite and the effect of the thickness of the molded part.

**Figure 9:**
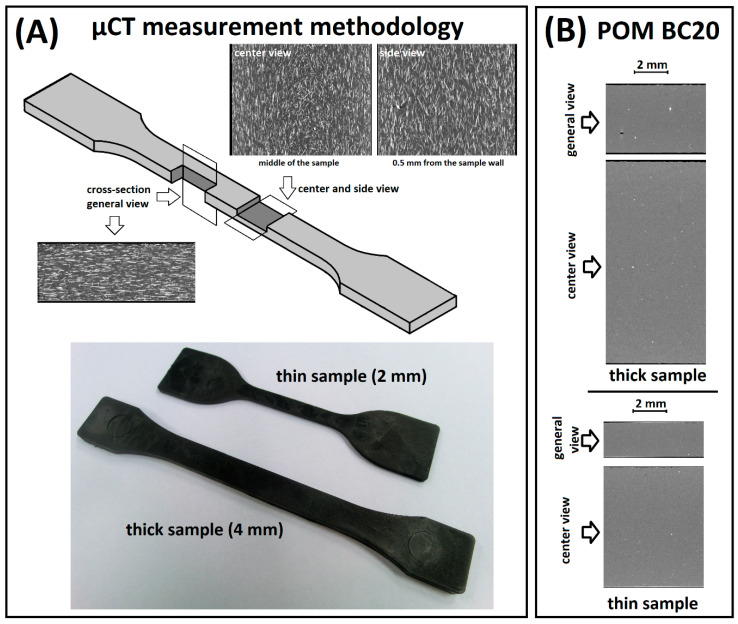
(**A**) The methodology of the μCT measurements, (**B**) homogenous structure of the POM BC20 samples.

Figure 9 presents a schematic diagram of the measurement methodology as well as a view of the homogeneous structure of the POM BC20% composite. It can be clearly seen that the structure anisotropy is impossible to assess in this case due to the small size and spherical shape of the BC filler.

**Figure 10:**
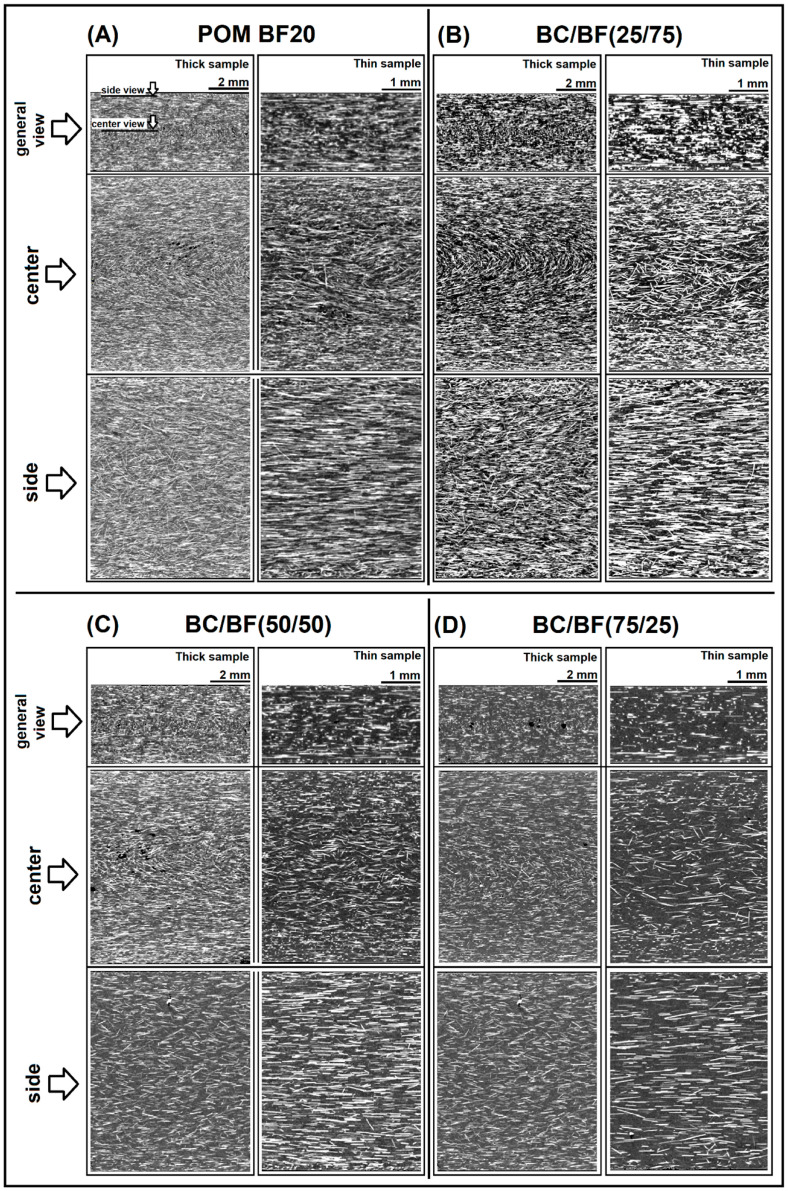
Orientation analysis of composite samples. Cross-section images from μCT measurements for: (**A**) reference POM BF20% sample; (**B**) POM(25/75); (**C**) POM(50/50), and (**D**) POM(75/25) composites.

**Figure 11:**
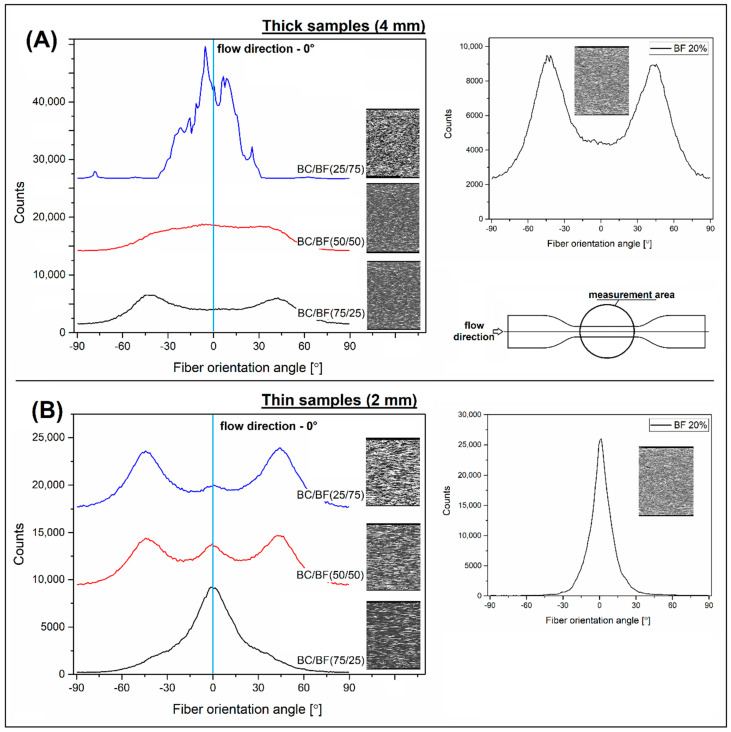
The comparison of the fiber orientation histograms of hybrid BC/BF composites: (**A**) for thick 4 mm samples, (**B**) for thin 2 mm samples. Separate histograms show the results for reference POM BF20% samples.

The addition of fibers dramatically changed the appearance of the structure, which is visible in Figure 10. The image comparison presents the cross-sections of 2 and 4 mm thick samples. Apart from the fiber orientation analysis shown in Figure 11, even a cursory analysis of cross-sections indicates a much greater fiber orientation for thin-walled samples (2 mm). A significant fiber orientation is particularly evident in the images taken close to the outer surface of the sample, where almost all the BF have a direction consistent with the sample’s main axis. This type of unidirectional arrangement is not recorded for thick samples (4 mm). The biggest differences are visible when comparing the side cross-section of the samples; for the prepared materials, the presented side view was made at a depth of 0.5 mm from the sample surface. Interestingly, a clear difference in the thickness of the core layer can be seen, which for thick samples is proportionally smaller.

Observations of thick samples also allowed us to clearly observe the fountain flow of material. The clearly parallel orientation of the fibers at the specimen wall gradually disappeared, so that in the central plane of the cross-section, a significant part of the fibers were arranged perpendicular to the direction of material flow, while the core layer structure was mostly isotropic. For thin sample, a fountain transition layer could not be distinguished. The boundary between the randomly oriented core structure and the unidirectional skin layer was very sharp. This type of behavior has been observed for glass fiber-reinforced samples [87]; however, the variable material factor was the content of reinforcement fibers (from 30% to 65%), not the hybridization as in the case of the discussed studies.

The addition of BC particles reduced the amount of BF fibers, which was also visible in the images obtained from the CT scans. It is worth noting that due to the low density in relation to fibers, the BC particles were not visible in the cross-sections of the samples, which indirectly facilitated observation of the orientation of the fibers themselves. The histograms shown in Figure 11 were prepared using image analysis of the side cross-sections of the samples; therefore, they reflect the orientation occurring in the skin layer. The purpose of the analysis was to verify the expected changes in BF fiber orientation, which was one of the reasons for the addition of BC particles.

As the results indicate, the reference sample structure for thin and thick POM BF 20% specimens was different. In the case of thin samples, the skin layer was definitely unidirectional, whereas for thick ones, the fiber orientation seemed to be more random. The graphs describe this situation in detail, where for thin samples the orientation angle of most fibers was close to 0°, which corresponds to the direction of flow of the polymer in the mold. Fiber orientation for thick samples was less homogeneous. Changes in the shape of histograms after the addition of BC particles also suggest slightly different behavior of the structure depending on the thickness of the product. For 4 mm samples, the addition of 25% BC particles increased fiber orientation, while the addition of 50% and 75% BC filler caused the desired effect in the form of fiber dispersion and randomization of the composite structure. The initial increase in the orientation may be associated with a change in the degree of interaction of reinforcement fibers; the introduction of spherical particles limited the phenomenon of the fibrous structure entanglement and as a result, increased the ability to form a unidirectional arrangement.

For thin 2 mm samples, the histograms of BF orientation indicate the high efficiency of the hybridization method. Even with the addition of BC at the level of 25%, the fiber orientation was disturbed; a similar effect was maintained at 50% BC, while for POM samples (75/25), an increasing level of orientation of the fibrous structure was observed. Despite a clear increase in the number of fibers oriented along the injection direction, the histogram also indicated a very wide distribution of fiber orientation directions in the structure, whereas for the POM BF20 reference sample, the arrangement of most fibers followed the polymer flow direction.

## 4. Conclusions

Most of the measurement results for composite samples indicate the positive effects of using BC particles as a powder filler. This material could be successfully used as a substitute for mineral fillers. In addition, the low density of the BC filler could lead to a reduction in the weight of the products produced. The direct comparison of the reference samples prepared with the addition of pure BC and BF indicates a reduction in density of around 8%. However, it is worth emphasizing that for the tested composites, the filler content is only 20 wt.%, which can be considered relatively low. The analysis of the mechanical test results showed a significant impact of the BF content on the stiffness of the composites. The reduction of the amount of fibers, due to the addition of BC particles, caused a decrease in the tensile modulus. However, other mechanical factors were largely independent of the BC content. The tensile strength values remained at a similar level for all prepared samples. The elongation at break and impact strength values remained at a similar level for all composites, however, a significant reduction of both parameters was noted when compared to the samples from the pure POM. Thermomechanical properties of composites were visibly improved compared to samples based on unmodified POM. Similar to the measurements of tensile modulus, the values of the storage modulus increased with the increasing share of BF in the structure. The stiffness improvement translated into large increases in HDT results, while for the POM BF20% sample, the heat resistance reached approximately 162 °C. For the rest of hybrid composites, the HDT was around 157–160 °C.

Results of the rheological measurements showed a decrease in viscosity due to the addition of BC; this fact may be associated with a certain amount of moisture in the BC structure (≈1%), and its presence during processing may lead to hydrolytic degradation of the POM matrix. Despite the visible changes in viscosity, the matrix degradation did not adversely affect the injection molding process, so it can be considered that the scale of this phenomenon is relatively small.

One of the key aspect of using hybrid reinforcement is the reduction of structure anisotropy, which is usually associated with the presence of fibrous fillers. Studies have shown the positive effect of the addition of BC particles; the unidirectional arrangement of BF is disturbed, which results in greater randomization of the composite structure.

## Figures and Tables

**Table 1:** The list of parameters of the extrusion and injection molding process.

Parameter	Unit	
**Extrusion**		
Temperature Profile	(C)	190 (nozzle)-190-190-190-185-185-180-180-175
Screw Speed	rpm	100
Feeding Rate	kg/h	3
**Injection Molding**		
Temperature Profile	(°C)	200 (nozzle)-200-200-190-180
Injection/Holding Pressure	(bar)	1050/600
Holding/Cooling Time	(s)	10/20
Clamping Force	(kN)	350
Screw Speed	(rpm)	300
Screw Back Pressure	(bar)	50
Mold Temperature	(°C)	80

**Table 2:** List of prepared composite types, sample formulations, results of the density measurements, and porosity/void content calculations.

Sample	Polyoxymethylene (POM)		Biocarbon (BC)		Basalt Fiber (BF)		Density (g/cm^3^)	Porosity * (%)
	(wt.%)	(vol%)	(wt.%)	(vol%)	(wt.%)	(vol%)		
POM pure	100		-		-		1.387 (±0.018)	1.59
POM BC20	80	82.4	20	17.6	-		1.407 (±0.017)	2.29
POM(75/25)	80	84.3	15	13.0	5	2.7	1.434 (±0.055)	2.44
POM(50/50)	80	85.8	10	8.7	10	5.5	1.474 (±0.017)	1.68
POM(25/75)	80	87.3	5	4.3	15	8.4	1.497 (±0.003)	2.11
POM BF20	80	88.5	-		20	11.5	1.527 (±0.004)	2.10

* for the purpose of porosity calculations, we used the results of helium pycnometer measurements; the density of BC was 1.612 g/cm^3^ and of BF, 2.653 g/cm^3^.

**Table 3:** The list of parameters of extrusion and injection molding processes.

Resin Type (Producer)	Filler Type	Tensile Modulus/Strength (MPa)
RTP 842 (RTP company)	Mineral filler	3170/53.4
Duracon TR-20 (Polyplastics)	Mineral Filler	4500/59
POM-90MC20 (PTS)	Mineral filler	3310/44.8
Tarnoform 300 GF4 (Grupa Azoty)	Glass fiber	7900/105
Hostaform C9021 BV1/20 (Celanese)	Glass fiber	7200/120
RTP 803 (RTP Company)	Glass fiber	6550/96.5
RTP 803 UV (RTP Company)	Glass fiber	8270/82.7
RTP 802 SI2 (RTP Company)	Glass fiber	5860/75.8
Lupital FC2020D (Mitsubishi)	Carbon fiber	17000/130
Duracon CH-20 (Polyplastics)	Carbon fiber	14000/144
RTP 883 TFE20 (RTP Company)	Carbon fiber	13800/103

**Table 4:** The comparison of DMTA analysis results, N factor calculations, and HDT measurements.

Sample	Thermomechanical Properties (DMTA/HDT)				
	Storage Modulus, G’ at 25 °C (°C)	Storage Modulus, G’ at 135 °C (%)	Degree of Entanglement N		HDT (0.455 MPa) (°C)
			25 °C	135 °C	
POM pure	1.12 × 10^9^	2.05 × 10^8^		-	135.5 (0.2)
**Composites**					
BC20%	1.72 × 10^9^	4.85 × 10^8^	1.15 × 10^5^	2.40 × 10^4^	154.8 (0.2)
BC25-BF75	1.82 × 10^9^	5.37 × 10^8^	1.22 × 10^5^	2.64 × 10^4^	157.2 (2.0)
BC50-BF50	1.92 × 10^9^	6.27 × 10^8^	1.29 × 10^5^	3.08 × 10^4^	158.8 (1.6)
BC75-BF25	1.93 × 10^9^	6.26 × 10^8^	1.29 × 10^5^	3.07 × 10^4^	160.5 (0.6)
BF20%	2.10 × 10^9^	7.72 × 10^8^	1.41 × 10^5^	3.79 × 10^4^	161.8 (0.1)

**Table 5:** Thermal properties of POM-based composites obtained during DSC measurements.

Sample	Thermal Properties			
	Enthalpy (J/g)	Melting Peak (°C)	Crystallinity (Content) (%)	Onset Temperature (Crystallization) (°C)
POM Pure	130.8	169.9	70.7	148.2
	**Composites**			
BC20%	100.8	167.9	68.1	148.5
BC25-BF75	103.0	168.1	69.6	148.3
BC50-BF50	103.4	168.6	69.9	147.9
BC75-BF25	105.0	169.0	70.9	147.9
BF20%	105.4	168.0	71.2	147.7
